# Cigarette Smoking and Human Gut Microbiota in Healthy Adults: A Systematic Review

**DOI:** 10.3390/biomedicines10020510

**Published:** 2022-02-21

**Authors:** Martina Antinozzi, Monica Giffi, Nicolò Sini, Francesca Gallè, Federica Valeriani, Corrado De Vito, Giorgio Liguori, Vincenzo Romano Spica, Maria Sofia Cattaruzza

**Affiliations:** 1 Department of Public Health and Infectious Diseases, Sapienza University of Rome, 00185 Rome, Italy; martina.antinozzi@uniroma1.it (M.A.); monica.giffi@uniroma1.it (M.G.); sini.1876851@studenti.uniroma1.it (N.S.); corrado.devito@uniroma1.it (C.D.V.); mariasofia.cattaruzza@uniroma1.it (M.S.C.); 2 Department of Movement Sciences and Wellbeing, University of Naples “Parthenope”, 80133 Naples, Italy; giorgio.liguori@uniparthenope.it; 3 Department of Movement, Human, and Health Sciences, University of Rome “Foro Italico”, 00135 Roma, Italy; vincenzo.romanospica@uniroma4.it

**Keywords:** gut microbiota, cigarette smoking, e-cigarettes, phylum diversity

## Abstract

The intestinal microbiota is a crucial regulator of human health and disease because of its interactions with the immune system. Tobacco smoke also influences the human ecosystem with implications for disease development. This systematic review aims to analyze the available evidence, until June 2021, on the relationship between traditional and/or electronic cigarette smoking and intestinal microbiota in healthy human adults. Of the 2645 articles published in PubMed, Scopus, and Web of Science, 13 were included in the review. Despite differences in design, quality, and participants’ characteristics, most of the studies reported a reduction in bacterial species diversity, and decreased variability indices in smokers’ fecal samples. At the phylum or genus level, the results are very mixed on bacterial abundance both in smokers and non-smokers with two exceptions. *Prevotella* spp. appears significantly increased in smokers and former smokers but not in electronic cigarette users, while Proteobacteria showed a progressive increase in *Desulfovibrio* with the number of pack-years of cigarette (*p* = 0.001) and an increase in *Alphaproteobacteria (p =* 0.04) in current versus never smokers. This attempt to systematically characterize the effects of tobacco smoking on the composition of gut microbiota gives new perspectives on future research in smoking cessation and on a new possible use of probiotics to contrast smoke-related dysbiosis.

## 1. Introduction

The pivotal role of the gut microbiota is now an unquestionable scientific assumption [1,2,3]. Several studies have demonstrated that it significantly contributes to maintaining the physiological equilibrium of the mucosal microenvironment, and it also interacts intimately with the intestinal immune system [1,2,3,4,5,6,7,8]. In particular, the microbiome is considered the “new” biomarker of human health because of its fundamental role in maintaining normal body physiology while developing and educating the immune system [1].

Indeed, the intestinal microbiota maintains the mucosal integrity, regulates the absorption of ingested food, and exerts a competitive inhibition by preventing invasion or colonization by any other potential pathogenic microorganism [2]. Microbial products, such as short chain fatty acids (SCFAs) and polysaccharide A, modulate immune homeostasis and local immune response towards pro-inflammatory or anti-inflammatory status [3]. 

The clinical importance of the microbiota in maintaining the homeostasis in the human body is clear, particularly considering its involvement in a wide spectrum of human diseases ranging from autoimmune [4] to metabolic and neurological disorders [5]. Recent discoveries confirm that it is even able to affect the pharmacological response to drugs [6]. Moreover, the microbial components can interact with local immune cells leading to functional changes also outside the gastrointestinal tract: it can cause alterations in the release of circulating cytokines, and it can influence immune cells in other body sites, such as the brain. In particular, the gut microbiota is reported to regulate the microglia in its development and functioning [3].

Nowadays, thanks to the availability of new molecular identification techniques based on 16S ribosomal RNA sequencing, it is known that about 30 different bacterial phyla and more than a thousand species coexist in the intestinal microenvironment [7]. Among the various phyla, Firmicutes and Bacteroidetes are undoubtedly predominant. Firmicutes, mostly consisting of Gram-positive bacteria of the genera Bacilli and Clostridia, is the most common phylum in the gastrointestinal tract, accounting for 11% to 43% of the microbial population. Bacteroidetes are Gram-negative, obligate anaerobic bacteria, with fermenting and non-fermenting properties [8].

Clinical and microbiological studies focused on the importance of species diversity for improving microbial community resilience, even considering that each individual tends to develop a specific microbial profile [9]. These profiles tend to be stable over time, even if they may be altered at any time by drugs, such as antibiotics [10] or lifestyle choices, including diet, physical activity, and smoking [11,12,13].

Cigarette smoking is a well-known risk factor for almost every disease; in particular, tobacco is an important part of the inflammation pathway in many diseases (e.g., asthma, Chronic Obstructive Pulmonary Disease (COPD, cancer). However, only recently have scientists started assessing its possible effects. not only as a pathogenetic player in multifactorial diseases, but also as a crucial element that can influence the human ecosystem.

The interest of the scientific community initially focused on the study of the upper airway’s microbiota, being the first mucosal contact of the body with smoke during inhalation [14]. 

In 2012, Garmendia et al. demonstrated that continuous exposure to tobacco smoke is associated with the presence of opportunistic pathogens, such as *Streptococcus pneumoniae*, *Haemophilus influenzae*, *Moraxella catarrhalis*, and *Streptococcus pyogenes* in the nasopharyngeal microbiota of smokers, whereas Beta-hemolitic Streptococci, *Peptostreptococcus* spp., and *Prevotella* spp. [15] were mainly found in non-smokers. Garmendia et al. also reported that cigarette smoke promotes pathogen colonization, whereas smoking cessation is associated with a reversion to the microflora detected in never smokers [15]. Subsequent research focused on the analysis of the relation between intestinal microbiota and smoke, hypothesizing a role of cigarette smoking in mediating the weight gain that commonly follows smoking cessation, connecting it with the development of diseases, such as inflammatory bowel disease (IBD) [16,17].

In murine models exposed to tobacco smoke, the intestinal microbiota is mostly composed by Firmicutes and Actinobacteria, less by Bacteroides and Proteobacteria; this profile was associated with weight gain during the observational period, even without changes in the murine diet [18].

Similar results were detected in obese humans, who express microbial profiles that are more efficient in the extraction of calories from ingested food [2].

Since this first piece evidence, further studies have tried to address the effects of cigarette smoking on gut microbiota composition. In 2018, Savin et al. [19] reviewed this, considering the intestinal and non-intestinal microbiome in humans and animals, both in physiological and pathological conditions. Through their findings they tried to give possible explanations for smoking-induced dysbiosis: “smoking reduces inflammatory pathways by decreasing phosphorylation of NFkB-P65, a key mediator in the NFkB inflammatory pathway [20] and was shown to alter levels of cytokines, such as CXCL2, IL-6, INF-γ, and TGF-β [18,21]. Smoking also generates reactive oxygen species (ROS) in the blood stream, resulting in oxidative stress [22,23]. Nevertheless, their findings suggested the necessity to carefully examine the interaction between smoking and microbiota on the development of intestinal and systemic diseases.

In 2021, Gui et al. reported that tobacco smoking has been associated with significant changes in gut bacterial taxa [24]. Indeed, smoking implies the assumption of more than 7000 toxic substances that could play a role in gut microbiota composition, however research to identify the specific influence of these toxic substances on gut microbiota is still ongoing.

Even electronic cigarette (e-cigarette) users are exposed to toxic substances, which can modify the inflammatory human response. In particular, the in vitro study by Lee et al. found that “exposure of endothelial cells to e-liquid, conditioned media induced macrophage polarization into a pro-inflammatory state, eliciting the production of interleukin-1β (IL-1β) and IL-6, leading to increased ROS” [25].

This systemic pro-inflammatory status might also have an impact on the gut microbiota composition, as suggested by available studies on the impact of e-cigarette use on animals’ gut microbiota and on oral microbiota composition in humans [26,27].

To date, the effects of smoking on gut microbiota have not been systematically evaluated, especially in humans. 

The aim of this systematic review is to analyze the available evidence concerning the relationship between cigarette smoking and human intestinal microbiota, in order to contribute to the characterization of the gut microbiota profile of healthy smokers and to highlight its potential impact on the host health status.

## 2. Materials and Methods

We conducted this systematic review in accordance with the Preferred Reporting Items for Systematic Reviews and Meta-Analyses (PRISMA) guidelines [28,29]. It has been registered with the International Prospective Register of Systematic Reviews (PROSPERO registration number: CRD42021169423).

### 2.1. Identification of Studies

We identified our MeSH terms and developed the search strategy using the PICO process (POPULATION–INTERVENTION–COMPARISON–OUTCOME) [30]. We searched all the available literature published until 7 June 2021 on three electronic databases: PubMed, Scopus, and Web of Science.

The search was conducted using the following keywords: (smok* OR cigarette* OR tobacco OR e-cig* OR “electronic cigarette” OR vaporizer*) AND ((microbio* OR bacteria* OR microbial OR flora OR microflora) AND (gut OR intestinal)) AND (English[lang]).

### 2.2. Eligibility Criteria 

All study designs (systematic review, randomized controlled trial, cohort study, case-control study, cross-sectional study, narrative review) on healthy adults with an age range of 18–65 years, no gender difference, and only tobacco smokers and e-cigarette users were considered. We evaluated only the intestinal microbiota collected on fecal samples and analyzed with genome sequencing of rRNA 16S.

The search was limited to the English language. 

Grey literature and studies considering second-hand smoke, air pollution, and upper airway microbiota were excluded.

### 2.3. Variability Indices

The primary aim was to assess the abundance of Phyla, the Phyla’s ratio, and the species’ variability measured through any variability index (mathematical measure) for alpha diversity and beta-diversity indices [31].

Among the alpha diversity, Shannon, Evenness, Simpson indices, Pielou’s evenness, Sobs, and Chao1 were used as “richness” and “evenness or equitability” indicators. 

Briefly, the Shannon index provides a statistic of diversity species assuming all species are represented in a sample and that they are randomly sampled, while the Simpson and Pielou indices are dominance indicators providing the description of species distribution [31,32]. Moreover, Sobs, Chao1, and Heip indices are mainly sensitive to the variation of rare species, could indicate rare OTUs [33]. 

Beta-diversity indices, such as Bray–Curtis dissimilarity or UniFrac, were used to evaluate the different structures of the communities between samples, both considering samples’ phylogeny (weighted UniFrac) and evaluating the presence/absence of genera in the samples (unweighted UniFrac) [34].

### 2.4. Primary and Secondary Level Screening

Three authors independently screened for relevance a total of 1217 articles by titles and abstracts using Jabref [35]. The first level of screening was based on the inclusion and exclusion criteria. In the second level of screening, studies indicated as relevant were subsequently reviewed as full-text. Disagreements were solved with third-party consultation. Authors reached a consensus for all included studies. 

### 2.5. Data Extraction 

Data were extracted using a standardized extraction table in Microsoft Excel and verified for completeness and accuracy by all authors. We collected information on study characteristics (author, country, year of publication, study design); methods of study (setting, population characteristics, timing of tobacco exposure); outcomes (abundance of phyla, variability index, phyla ratios); and the main results.

### 2.6. Quality Assessment 

We assessed the methodological quality of included studies by using the following scales. The “Methodological index for non-randomized studies” (MINORS) [36] for non-randomized studies; it is composed of eight items for non-randomized studies and four more items in the case of comparative studies, it is based on a scoring system from zero to two, so that zero is “not reported”, one is “reported but inadequate”, and two is “reported and adequate”. The global ideal score is at least 16 for non-comparative studies and 24 for comparative studies. 

The Joanna Briggs Institute Critical Appraisal tool [37] was used for cross-sectional studies; it consists of a scoring protocol from one to eight, based on the presence, absence, how unclear the information was, or the non-applicability of the item. Studies were considered of good/high quality when a total score of 5/8 was reached in the quality assessment, whereas a lower score was classified as poor quality.

## 3. Results

**Figure 1:**
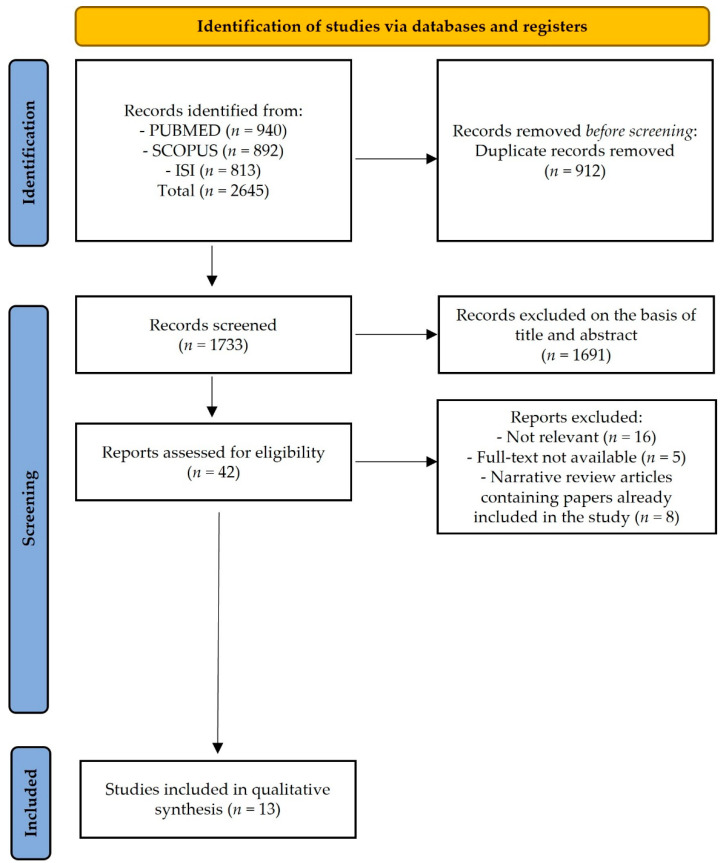
PRISMA 2020 flow diagram for study selection.

The bibliographic search yielded 2645 records, of which 2603 were excluded after the removal of duplicates and screening by title and abstract. A total of 29 records were excluded after reviewing the full text, leaving 13 studies for inclusion in the review (Figure 1) [38,39,40,41,42,43,44,45,46,47,48,49,50]. 

### 3.1. General Characteristics of the Studies 

The main features of the included studies are summarized in Table 1. 

The 13 studies included were published between 2013 and 2021. Studies were conducted in the United States [38,39,40,41,42,43], China [44,45,46], Switzerland [47], Korea [48], Saudi Arabia [49], and Japan [50]. 

Twelve had a cross-sectional design [38,39,40,41,42,43,44,45,46,48,49,50], one study had a controlled prospective design [47]. The sample size ranges from N = 20 to N = 803. 

Four out of twelve cross-sectional studies compared the microbiota composition between smokers (>10 cigarettes/day or daily use of e-cig for minimum 6 months) and non-smokers [28,30,33,39]; in three studies, researchers differentiated between current-smokers, former-smokers, and non-smokers [42,43,48].

All studies considered cigarette smoking as a source of tobacco, except for two where both cigarette smoking and e-cigarette smoking were considered [39,41]. 

In their controlled prospective study, Biederman et al. analyzed stool samples of healthy smoking human subjects undergoing controlled smoking cessation during a 9-week observational period compared with two control groups, consisting of ongoing smoking and nonsmoking subjects [47]. 

Ten out of thirteen studies assessed dietary habits of participants [40,41,42,44,45,46,47,48,49,50]. In three, results were adjusted for diet [40,41,45], but in one a relationship was found between yogurt assumption and increased diversity in intestinal microbiota [45] and in another coffee consumption was related to higher concentration of Bacteroides species [49].

### 3.2. Diversity Analysis 

The results of the selected studies are summarized in Table 2. 

All included studies but one [40] assessed microbial diversity. In particular, ten studies [38,39,41,42,43,44,45,46,47,48] calculated just alpha diversity and seven of them [38,42,45,46,47,48] also beta diversity. Only Harakeh et al. used the Chao1 Index to estimate diversity from abundance data [49].

Nine out of thirteen studies used the Shannon index to assess alpha diversity [38,39,41,43,44,45,46,48,49], one study also calculated the Pielou index [45] and another analyzed phylogenetic diversity within group diversity [47]. 

A statistically significant reduction of the Shannon index among tobacco smokers was shown in four studies [39,41,45,48] and just one study found a significant reduction of the Pielou index [45]. 

A statistically significant reduction of the Shannon index was also found in e-cigarette users in the study by Curtis et al. [39]. However, a decreasing trend of the Shannon index, both among tobacco [28,44] and among e-cigarettes smokers [41], was found in studies that did not produce statistically significant results.

On the other hand, it is interesting to note that Biedermann et al. found an increase in the alpha diversity after smoking cessation [47].

When beta diversity was considered, Biedermann et al. found a statistically significant difference between the UniFrac distance in subjects undergoing smoking cessation, comparing the time points prior to and after the smoking cessation intervention [47]. Another study found similar results between tobacco smokers and non-smokers [39]. These results were confirmed by Lee et al., who showed statistically significant beta diversity, using Jaccard-based diversity analysis, between former smokers and current smokers and between never smokers and current smokers [48]. 

Finally, Chen et al. found that tobacco use showed a trend toward association with the microbiota using UniFrac distance [38].

### 3.3. Methodological Quality of the Studies

The quality assessment for all included studies is summarized in Supplementary Tables S1 and S2 and in Supplementary Figure S1.

In general, according to Joanna Briggs Institute Critical Appraisal tools, the quality of cross-sectional studies included in the review was good, since 6 out of 7 studies obtained a score higher than 5/8, while just 1 scored 4/8; all studies satisfied the items 4,5,7 and 8 of the JBI tool. The item 6, concerning the application of strategies to deal with confounding factors, though identified by all authors, was the most neglected.

The quality of the only controlled prospective study, according to the “Methodological index for non-randomized studies” (MINORS), was moderate and scored 17 out of 24 points. 

### 3.4. Cigarette Smokers, Electronic Cigarette Users, Former-Smokers, and Never-Smokers

The cross-sectional studies of Kato et al. and Nolan-Kenney et al. find a significant increase in Proteobacteria (at the genus level) in the smokers’ sample [40,43]. Specifically, a progressive increase in *Desulfovibrio* DNA, related to the number of pack-years of cigarette smoking (*p* = 0.001) [40], and in *Alphaproteobacteria* [43] were found. 

A significant increase in *Bacteroides* was found by Ishaq, Lee, Zhang, Lin, and Harakeh et al. [44,45,46,48,49], in contrast with a significant decrease found by Curtis et al. (valid both for tobacco and e-cigarette smokers), Stewart, and Biedermann et al. [39,41,47]. 

Concerning the Bacteroidetes phylum: the characteristics of *Prevotella* are unexpected, according to Curtis’ team results, and *Prevotella* seems to have a different behavior depending on the tobacco source, with a significant increase in tobacco smokers and a significant decrease in e-cigarette smokers [39]. A significant increase in *Prevotella* in smokers in comparison to controls was also found by Stewart et al. and Prakash et al. [41,42]. 

In particular, Stewart et al. analyzed tobacco and e-cigarette smokers, finding two different profiles at the genus level: increased *Prevotella* (*p* = 0.006) and decreased *Bacteroides* in tobacco smokers [41]. Specifically, *Prevotella* had significantly increased relative abundance in tobacco smokers compared to controls (*p* = 0.008) and e-cigarette users (*p* = 0.003), but no difference between e-cigarette users and controls (*p* = 0.99) was found. Meanwhile, *Bacteroides* showed significantly decreased relative abundance in tobacco smokers compared to controls (*p* = 0.017) and e-cigarette users (*p* = 0.003), but no difference between e-cigarette users and controls (*p* = 0.684).

In 2013, in a prospective controlled study on smoking cessation, Biedermann et al. reported that smokers showed less *Firmicutes* and *Actinobacteria* than non-smokers, which tend to increase after smoking cessation.

At the same time, the proportion of *Proteobacteria* and *Bacteroidetes*, higher in smokers than non-smokers, tends to decrease after smoking cessation [47]. The results of Lee et al., Shima et al., and Lin et al. are in accordance with this [46,48,50]. 

Specifically, Lee’s team found a significantly decreased proportion of *Firmicutes* in smokers than former-smokers and never-smokers (respectively increasing from the status of smokers to never-smokers, *p* = 0.015) and a significantly increased proportion of *Bacteroidetes* in smokers than former and never-smokers (decreasing from current smokers to never-smokers, *p* = 0.047) [48].

### 3.5. Smokers before and after Smoking Cessation Intervention

The article of Biedermann et al. is the only controlled prospective study of this systematic review [47]. Analyzing the results of this eight-week smoking cessation intervention, they found an increase of sequences from *Firmicutes* and *Actinobacteria* and a simultaneous decrease of *Proteobacteria* and *Bacteroidetes* fractions after smoking cessation. These changes were observed exclusively in the intervention group and particularly between screening phase (t1) and four weeks after smoking cessation (t2) [47].

Statistical significance was found in the increase in *Firmicutes* (*p* = 0.027) and *Actinobacteria* (*p* = 0.014) as well as in the decrease of *Proteobacteria* (*p* = 0.041) between t1 and t2, but not for the decrease of *Bacteroidetes* (*p* = 0.109).

These changes were enhanced at t3, eight weeks after smoking cessation, even though the composition of phyla between t2 and t3 remained strikingly similar with the exception of *Bacteroidetes*, leaning to both a relatively brisk (within four weeks) and durable (eight-week interval) effect of smoking cessation on microbial composition. In contrast, in the control groups, there was no significant change in the microbial composition at the phylum level.

Alpha diversity was shown to be substantially higher four weeks after smoking cessation compared to the samples obtained whilst smoking. After 8 weeks there still was a tendency towards increased diversity levels compared to baseline. In the control groups, both diversity indices were relatively stable.

In conclusion, Unifrac distance was analyzed as a measure of difference in the phylogenetic lineages in different environments. Biedermann and his team determined the highest Unifrac distance in subjects undergoing smoking cessation at t1 in comparison with t2 and t3, whereas no difference was observed both in the intervention and control groups after intervention (t2 vs. t3). 

## 4. Discussion

This review was aimed at evaluating the available evidence on the interaction between cigarette smoking and intestinal microbiota of healthy humans. Although the examined studies differed in design, quality, and participants’ characteristics, it is of concern that the majority of them reported lower levels of bacterial species diversity in smokers’ fecal samples. This evidence is in accordance with previous results obtained analyzing oral and gut microbiome, coming from animal and human models [24,51]. Despite the limited number of dedicated studies, even the use of e-cigarettes seems to be associated with a low gut microbiota variability [39,41].

Conversely, inconsistent results were reported for *Firmicutes* and *Bacteroidetes* at the phyla or genus level. In particular, the genus *Bacteroides* was reported to be mainly represented in smokers by four studies [44,45,46,49] and in non-smokers by two other studies [39,41]. *Prevotella spp.* was found to be highly abundant in cigarette smokers [39,41,42] but lower in e-cigarette smokers [41]. It should be noted that these results could have been affected by confounding factors. Only four studies adjusted their results for identified confounders [40,42,43,50].

The underlying mechanism linking cigarette smoking with intestinal microbiota dysbiosis is largely unknown. Several compounds and mechanisms have been proposed that may regulate this interaction [24]. 

Cigarette smoke contains many toxic substances, including polycyclic aromatic hydrocarbons (PAHs), aldehydes, nitrosamines, and heavy metals, which are inhaled into the lungs. These substances may reach the gastrointestinal tract and induce microbiota dysbiosis via different mechanisms, such as antimicrobial activity or regulation of the intestinal microenvironment [24,52].

Exposure to smoke components can benefit some bacteria populations by elevating the intestinal pH or decreasing the production of organic acids, enabling some species to thrive, and cause intestinal microbiota dysbiosis [53,54].

Changes in the concentration of bacteroides, which normally constitute about 25% of all gut microbiota and provide amino acids and vitamins from dietary proteins, seem to modulate gut production of amino acids (serotonin, catecholamines, glutamate), with a possible role in the alteration of vagal nerve transmissions to the brain [55]. Polycyclic aromatic hydrocarbons, which result mainly from the thermal cracking of organic resources and incomplete burning of organic material at low temperatures, may cause various diseases due to their toxicity, mutagenicity, and carcinogenicity. Intestinal microbiota can transform these compounds into non-hazardous or less toxic substances through fermentation [56]. However, evidence suggests that excessive ingestion of these substances may significantly alter the diversity and abundance of the intestinal microbiota, causing moderate inflammation and increasing the penetrability of intestinal mucosa [57].

Cigarette smoke contains high levels of toxic volatile organic compounds (VOCs), such as benzene. Some studies have shown that benzene may alter the overall structure of intestinal microbiome [58].

Acetaldehyde, a low-molecular-weight aldehyde, is a highly reactive substance that may cause different diseases, such as liver injury and gastrointestinal cancers. Many intestinal bacteria can convert acetaldehyde into ethanol through fermentation, which can lead to the overgrowth of relevant bacteria species [59]. Furthermore, acetaldehyde increases the permeability of the intestinal tract, allowing microorganisms and endotoxin to cross the intestinal mucosal barrier. Acetaldehyde also induces endotoxemia, with subsequent injuries to liver and other organs, intestinal inflammation, and rectal carcinogenesis [60]. In addition, acetaldehyde and reactive oxygen species induce neutrophil infiltration and consequent release of tissue-damaging compounds, which cause translocation of intestinal microbiota [61].

The main toxic gases contained in tobacco smoke enter into blood through alveolar exchange, which affects O_2_ transport, decreases blood pH, and induces systemic inflammation and diseases. Exposure to carbon monoxide in particular alters the intestinal microbiome by favoring bacterial species that express molecules involved in iron acquisition [62]. 

Moreover, cigarette smoke contains heavy metals (such as cadmium, arsenic, chromium, iron, mercury, nickel) which may be ingested and cause intestinal microbiota dysbiosis affecting the transport, oxidative, and inflammatory status of gut epithelium [63,64].

The human gut microbiome has a pivotal role in regulating inflammatory pathways taking part in the so-called gut-brain and gut-lung axes and there is evidence that pulmonary disorders may be implicated in the development of intestinal diseases. Patients with chronic lung diseases, whose pathogenesis is strictly related to cigarette smoking, have a higher prevalence of intestinal diseases, such as Intestinal Bowel Disease and Intestinal Bowel Syndrome [65,66]. Nicotine, or its metabolites, reduces gut microbial diversity and it worsens the symptoms in patients with Crohn’s disease [67]. There is much evidence that gut-residing microorganisms interact with the immune system, linking gut dysbacteriosis with inflammation progression and tobacco-related illnesses (e.g. asthma, COPD). Furthermore, tobacco is a well-known factor related to the release of inflammatory cytokines, which are a milestone for the development of diseases, such as cancer [1,3,68]. Similarly, the vapor of e-cigarettes seems to contribute to exposure to toxic aldehydes (e.g., formaldehyde and acrolein) released by thermal decomposition of the major vehicle components of e-cigarette e-liquids (propylene glycol and glycerol) and flavorings [69].

Dysbiosis of intestinal microbiota is also closely associated with skin diseases, such as acne, psoriasis, and atopic dermatitis. Cigarette smoking may lead to intestinal microbiota dysbiosis through the skin-gut axis. Skin inflammation might contribute to intestinal disorders through immunologic regulations and shifts in the microbiota composition [70]. 

For all these reasons, research on humans is needed to better clarify these mechanisms and to provide possible methods to counteract their effects after smoking cessation.

This review has limitations. First, selected studies show important differences in sociodemographic characteristics (two studies enrolled only males) and smoke exposure of participants (mainly self-reported), which limited the comparison and may affect the consistency of results. 

Furthermore, the studies differed in quality, and the main quality item involved was related to the lack of strategies to take account of the confounding factors, which weakened the strength of the findings. In particular, only a few studies considered the possible interference of diet on smoking-related effects on gut microbiota composition.

However, this review represents the first attempt to characterize, systematically, the effects of tobacco smoking on gut microbiota composition in healthy humans and it opens new perspectives for future research about strategies of smoking cessation and the possible role of probiotics to counteract smoke-related dysbiosis.

## 5. Conclusions

The evidence shows that intestinal microbiota dysbiosis is closely associated with intestinal and extra-intestinal diseases. Smoking seems to alter gut microbiota composition, inducing dysbiosis. However, the mechanisms by which the smoke toxicants alter human intestinal microbiota are not yet clearly defined, as well as the influence of the type of cigarettes (traditional and electronic) and the conditions of smoking (indoor/outdoor, active/passive smoking, amount of cigarettes/day, etc.) on the impact of these substances. 

Maintaining the balance of intestinal microbiota represents a new possibility for therapeutic approaches to smoking-related diseases. Further research is needed in this direction. 

## Figures and Tables

**Table 1:** Characteristics of the selected studies.

Author, Country, Year [Ref]	Study Design	Sample Characteristics°	Type of Cigarettes	Amount of Exposure - Assessment	Methodology	Statistical Adjustments	Diet	Quality of the Study
Biedermann, Switzerland, 2013 [47]	prospective, controlled	N = 20 (20 M; 18–60 years) 10 smokers undergoing cessation, 5 control smokers, 5 non-smokers	cigarettes	≥10 cigarettes/day - breath CO monitor	variable regions V1–V2 of the 16S rRNA gene sequencing	not adjusted	assessed: BMI decreased in smokers without diet modifications	MINORS: 17/24
Chen, USA, 2016 [38]	cross-sectional	N = 118 (60 F, 58 M; 20–79 years) 17 smokers	cigarettes	not specified - self reported	16S rDNA–targeted sequencing	not adjusted	not assessed	JBI: 6/8
Curtis, USA, 2019 [39]	cross-sectional	N = 30 (3 F, 27 M)10 cigarette smokers (37 ± 3), 10 e-cigarette smokers (30 ± 3), 10 non-smokers (32.2 ± 2)	cigarettes, e-cigarettes	cigarette smokers: ≥10 cigarettes/day; e-cigarette smokers: 6 months - self reported	16s rRNA PCR	not adjusted	not assessed	JBI: 4/8
Harakeh, UAE, 2020 [49]	cross-sectional	N = 104 (54 F, 50 M; 24 ± 7.7) 19 smokers	not specified	not specified - self-reported	V3 and V4 regions of the 16S rRNA gene using Miseq technology	not adjusted	assessed for coffee consumption smokers who consumed coffee had higher concentrations of *Bacteroides thetaiotaomicron* followed by *B. massiliensis*	JBI:7/8
Ishaq, China, 2017 [44]	cross-sectional	N = 20 (20 M; 35–50 years) 14 smokers, 6 non-smokers	cigarettes	10 years - self-reported	V3 region of the 16S rRNA gene sequencing	not adjusted	assessed but not analyzed	JBI: 6/8
Kato, USA, 2010 [40]	cross-sectional	N = 62 (26 F, 36 M; ≥48 years or <48 with polyps) N of smokers not specified	cigarettes	not specified - Self-reported	16S rRNA real time PCR	adjusted for diet, physical activity, number of pack-years of cigarette smoking, BMI	analyzed; does not affect smoking effect	JBI: 8/8
Lee, Korea, 2018 [48]	cross-sectional	N = 758 (758 M) 203 smokers (45.7 ± 8.2), 267 former smokers (47.2 ± 8.5), 288 never smokers (44.2 ± 9.1)	cigarettes	never-smokers: <100 cigarettes/lifetime; former-smokers: ≥100 cigarettes/lifetime and no smoke in the last 1 month; current-smokers: ≥100 cigarettes/lifetime and smoke in the last 1 month - self reported	V3 and V4 regions of the 16S rRNA gene sequencing	subjects who had taken antibiotics, probiotics, and cholesterol-lowering medication, were excluded	assessed but not analyzed	JBI: 7/8
Lin, China, 2020 [46]	cross-sectional	N = 116 (116 M) 14 non-smoking and non-drinking (57.21 ± 17.40 years), 31 smoking only (49.84 ± 11.55), 28 drinking only (50.07 ± 10.7), 43 smoking and drinking combined (47.44 ± 9.74)	cigarettes	not specified - self-reported	V3-V4 region of the 16S rRNA gene sequencing	not adjusted	alcohol drinking	JBI: 6/8
Nolan-Kenney, USA, 2019 [43]	cross-sectional	N = 249 (147 F, 102 M; 48.6 ± 7.9) 151 never smokers, 36 former smokers, 62 current smokers	cigarettes and bidis (Bangladesh locally produced cigarette)	bidis calculated in packs per day = number of sticks smoked per day divided by 20 - self-reported	V3-V4 region of the 16 s rRNA gene sequencing	adjusted for sex, age, BMI, betel quid use, and education	not assessed	JBI:7/8
Prakash, USA, 2021 [42]	cross-sectional	N = 803 (507 F, 296 M; 38–87 years) 543 never smokers, 181 former smokers, 79 current smokers	cigarettes	current smokers: daily use of ≤10 cigarettes N = 41; daily use of >10 cigarettes N = 37 - self-reported	V4 region of the 16S rRNA gene sequencing	adjusted for age, sex, race, BMI, and fiber	fiber intake assessed	JBI: 7/8
Shima, Japan, 2019 [50]	cross-sectional	N = 366 subjects (181 F, 185 M; 40.0 ± 11.0 years) 312 non-smokers, 54 smokers	Not specified	not specified - self-reported	reverse-transcription-quantitative polymerase chain reaction (RT-qPCR)	adjusted for age, sex, BMI, and frequency of alcohol, exercise, and fermented milk consumption	assessed fermented milk consumption	JBI: 7/8
Stewart, USA, 2018 [41]	cross-sectional	N = 30 (2 F, 28 M; 24–45 years) 10 cigarette smokers, 10 e-cigarette smokers, 10 non-smokers	cigarettes, e-cigarettes	daily use of e-cigarette for min 6 months; ≥10 cigarettes/day- self-reported	V4 regions of the 16S rRNA gene sequencing	not adjusted	assessed, no differences among groups	JBI: 7/8
Zhang, China, 2019 [45]	cross-sectional	N = 131 (51 F, 80 M; 22–69 years)	cigarettes	not specified - self reported	V3 and V4 regions of the 16S rRNA gene sequencing	not adjusted	assessed; yogurt+ have greater diversity	JBI: 8/8

°N = total sample size, F = number of females and M = number of males; age (range or mean +/− SD), characteristics of subgroups.

**Table 2:** Main findings related to gut microbiota variability and composition in smokers and former smokers vs. non-smokers from the selected studies.

Author, Year [Ref]	Variability	Firmicutes	Bacteroidetes	Actinobacteria	Proteobacteria	Tenericutes
Biedermann, 2013 (after smoking cessation) [47]	↑* UniFrac distance ↑* α-diversity in subjects undergoing smoking cessation	↑*	↓*	↑*	↓*	///
Chen, 2016 [38]	↓ Shannon index ↓ Unifrac distance	///	///	///	///	///
Curtis, 2019 [39]	↓* Shannon index ↑* Unifrac distance in tobacco smokers	/// ///	↑* *Prevotella* ↓* *Bacteroides* (in tobacco smokers);	/// ///	/// ///	/// ///
	↓* Shannon index in e-cigarette users		↓* *Prevotella* ↓ *Bacteroides* (in e-cigarette users)			
Harakeh, 2020 [49]	↔ Chao1/Shannon indices	↑ *Lactobacillus amylovorus*	↑* Bacteroides (↑ *B. thetaiotaomicron*)↓* Fusobacteria & Tenericutes	///	///	///
Ishaq, 2017 [44]	↓ Shannon index	↓ (*Lactobacillus* and *Clostridium leptum* subgroup)	↑* (*Bacteroides vulgatus*)	↓ (*Bifidobacterium*)	///	///
Kato 2010 [40]	///	///	///	///	↑*(Desulfovibrio)	///
Lee, 2018 [48]	↓* Shannon index ↓ Unifrac distance	↓*	↑*	///	↓*	↑*
Lin, 2020 [46]	↔ Sobs/Shannon/ Heip indices	↓* *Firmicutes* including several genus *Phascolarctobacterium*, Ruminococcaceae_UCG-002, Ruminococcaceae_UCG-003, andRuminiclostridium_9.	↑* *Bacteroides* (smoking pack-year)	↑* Actinomyces	///	///
Nolan-Kenney, 2019 [43]	↔ Simpson/Shannon indices	↑* Erysipelotrichi-to-Catenibacterium, Peptostreptococcaceae, Mitsuokella	///	↑* Slackia, *Collinsella*	↑* Alphaproteobacteria	///
Prakash, 2021 [42]	↓* Bray–Curtis dissimilarity in former smokers than never and current smokers	↓* Lachnospira ↑* Veillonellaceae in current and former smokers	↑*Prevotella in current and former smokers	///	///	↓* in current and former smokers
Shima, 2019 [50]	↓* total bacterial count ↓ Clostridium leptum in smokers	↓* Enterococcus	///	///	///	///
Stewart, 2018 [41]	↓* Shannon index in tobacco smokers	/// ///	↑* Prevotella ↓* Bacteroides	///	///	///
	↓ Shannon index in e-cigarette users		///	///	///	///
Zhang, 2019 [45]	↓* Shannon index ↓* Pielou index	///	↑*Bacteroides	///	///	///

↑= increase; ↓=decrease; ↑*= statistically significant increase; ↓*= statisticlly significant decrease; ↔ = no differences between groups; /// = not reported.

## Data Availability

No new data were created or analyzed in this study. Data sharing is not applicable to this article.

## Supplementary Materials

The following are available online at https://www.mdpi.com/article/10.3390/biomedicines10020510/s1. Table S1: Quality assessment of cross-sectional studies according to JBI tools; Table S2: Methodological items for non-randomized studies (MINORS)#-BIEDERMANN 2013; Figure S1: Quality assessment of cross-sectional studies.
