# Supplementary material for: Cigarette Smoking and Human Gut Microbiota in Healthy Adults: A Systematic Review
Source: Biomedicines. 2022 Feb 21;10(2):510. doi: 10.3390/biomedicines10020510 (PMC8962244; doi:10.3390/biomedicines10020510)
Supplement: Supplementary file 1 [file biomedicines-10-00510-s001.zip › STable 1.pdf]

Studies												Items
Chen, 2016	Curtis, 2018	Ishaq, 2017	Kato, 2010	Lee, 2018	Stewart, 2018	Zhang, 2019	Shima, 2019	Prakash, 2021	Nolan-	Harakeh,	Lin, 2020	
☺	☹	☺	☺	☺	☺	☺	☺	☺	☺	☺	☺	1.Were the criteria for inclusion in the sample clearly defined?
☺		☺					☺	☺		☺	☺	2.Were the study subjects and the setting described in detail?
☺		☹							☺	☺	☺	3.Was the exposure measured in a valid and reliable way?
☺	☺	☺					☺	☺	☺	☺	☺	4.Were objective, standard criteria used for measurement of the condition?
☺	☺	☺					☺	☺	☺	☺	☺	5.Were confounding factors identified?
☹	☹	☹		☹	☹		☺	☺	☺			6.Were strategies to deal with confounding factors stated?
☺	☺	☺		☺	☺		☺	☺	☺	☺	☺	7.Were the outcomes measured in a valid and reliable way?
☺	☺	☺		☺	☺		☺	☺	☺	☺	☺	8.Was appropriate statistical analysis used?

Supplementary Table S1. Quality assessment of cross-sectional studies according to JBI tools.
