# Supplementary material for: Cigarette Smoking and Human Gut Microbiota in Healthy Adults: A Systematic Review
Source: Biomedicines. 2022 Feb 21;10(2):510. doi: 10.3390/biomedicines10020510 (PMC8962244; doi:10.3390/biomedicines10020510)
Supplement: Supplementary file 1 [file biomedicines-10-00510-s001.zip › STable 2.pdf]

Items	Score
1. **A clearly stated aim**: the question addressed should be precise and relevant in the light of available literature	2
2. **Inclusion of consecutive patients**: all patients potentially fit for inclusion (satisfying the criteria for inclusion) have been included in the study	
during the study period (no exclusion or details about the reasons for exclusion)	0
3. **Prospective collection of data**: data were collected according to a protocol established before the beginning of the study	
4. **Endpoints appropriate to the aim of the study**: unambiguous explanation of the criteria used to evaluate the main outcome which should be in	2
accordance with the question addressed by the study. Also, the endpoints should be assessed on an intention-to-treat basis.	
5. **Unbiased assessment of the study endpoint**: blind evaluation of objective endpoints and double-blind evaluation of subjective endpoints.	2
Otherwise the reasons for not blinding should be stated.	
6. **Follow-up period appropriate to the aim of the study:** the follow-up should be sufficiently long to allow the assessment of the main endpoint	0
and possible adverse events	1
7. **Loss to follow up less than 5%**: all patients should be included in the follow up. Otherwise, the proportion lost to follow up should not exceed	2
the proportion experiencing the major endpoint	0
8. **Prospective calculation of the study size:** information of the size of detectable difference of interest with a calculation of 95% confidence interval,	
according to the expected incidence of the outcome event, and information about the level for statistical significance and estimates of power when	2
comparing the outcomes	2
9. **An adequate control group**: having a gold standard diagnostic test or therapeutic intervention recognized as the optimal intervention according	
to the available published data	2
10. **Contemporary groups:** control and studied group should be managed during the same time period (no historical comparison)	
11. **Baseline equivalence of groups**: the groups should be similar regarding the criteria other than the studied endpoints. Absence of confounding	2
factors that could bias the interpretation of the results	
12. **Adequate statistical analyses**: whether the statistics were in accordance with the type of study with calculation of confidence intervals or	TOT.
relative risk.	17/24
[#] The items are scored 0 (not reported), 1 (reported but inadequate) or 2 (reported and adequate).<br>The global ideal score being 16 for non-comparative studies and 24 for comparative studies.	

Supplementary Table S2. Methodological items for non-randomized studies (MINORS)[#] - BIEDERMANN 2013.
