# Supplementary figures and images for: Cigarette Smoking and Human Gut Microbiota in Healthy Adults: A Systematic Review
Source: Biomedicines. 2022 Feb 21;10(2):510. doi: 10.3390/biomedicines10020510 (PMC8962244; doi:10.3390/biomedicines10020510)

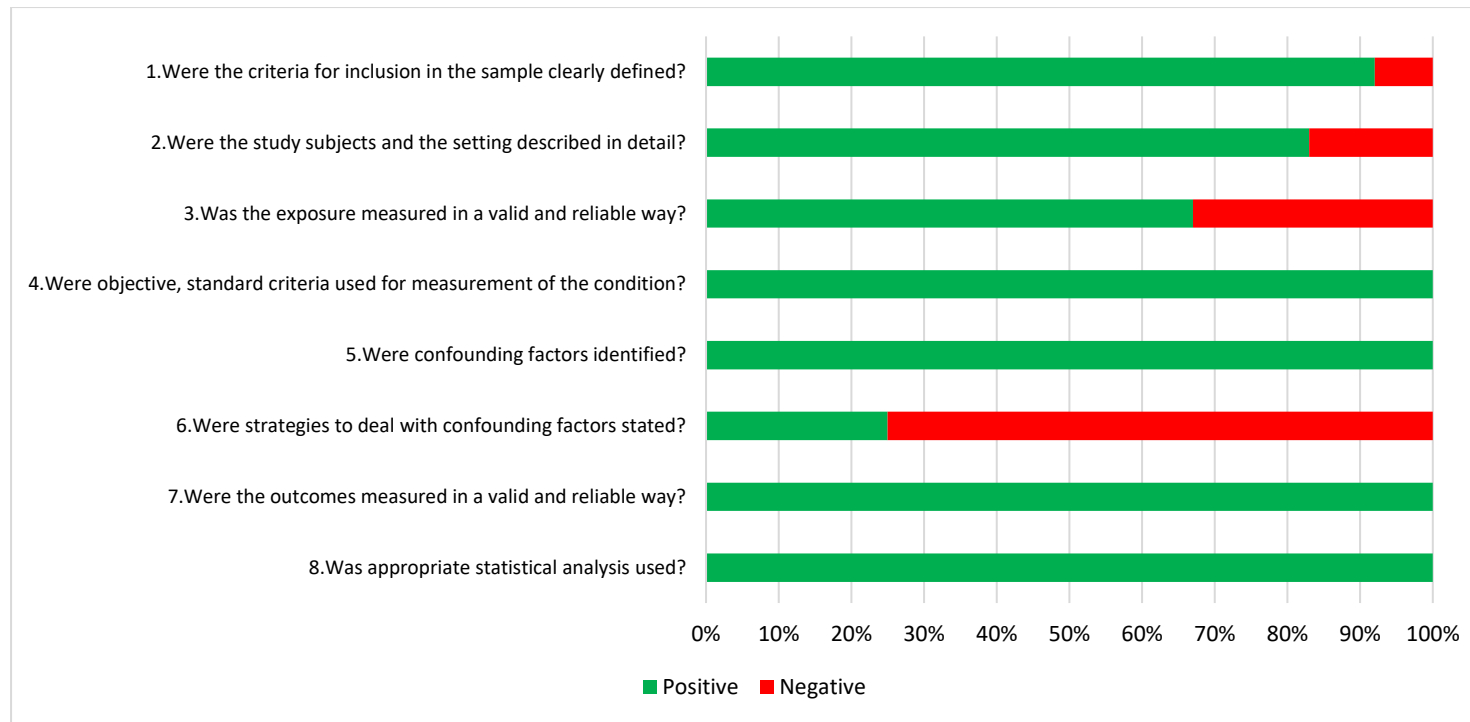

**Supplementary Figure S1.** Quality assessment of cross-sectional studies.

Supplement: Supplementary file 1 [file biomedicines-10-00510-s001.zip › SFigure 1.pdf]
